# Trust (Poetry)

**Published:** 1882-03

**Authors:** John G. Whittier


### TRUST.

A picture memory brings to me;
I look beyond the years, and see
Myself upon my mother's knee.

I feel her gentle hand restrain
My selfish moods, and know again
A child's blind sense of wrong and pain.

But wiser now, a man gray grown,
My childhood's needs are better known,
My mother's chastening love I own.

Gray grown, but in our Father's sight
A child still groping for the light
To read His works and ways aright.

I bow myself beneath His hand;
That pain itself for good was planned
I trust, but cannot understand.

I fondly dream it needs must be
That, as my mother dealt with me,
So with His children dealeth He.

I wait, and trust the end will prove
That here and there, below, above,
The chastening heals, the pain is love!

—*John G. Whittier.*

